# Dramatically decreased magnetoresistance in non-stoichiometric WTe_2_ crystals

**DOI:** 10.1038/srep26903

**Published:** 2016-05-27

**Authors:** Yang-Yang Lv, Bin-Bin Zhang, Xiao Li, Bin Pang, Fan Zhang, Da-Jun Lin, Jian Zhou, Shu-Hua Yao, Y. B. Chen, Shan-Tao Zhang, Minghui Lu, Zhongkai Liu, Yulin Chen, Yan-Feng Chen

**Affiliations:** 1 National Laboratory of Solid State Microstructures and Department of Materials Science and Engineering, Nanjing University, Nanjing 210093, China; 2 National Laboratory of Solid State Microstructure and Department of Physics, Nanjing University, Nanjing 210093, China; 3 School of Physical Science and Technology, ShanghaiTech University, Shanghai 200031, China; 4 State Key Laboratory of Low Dimensional Quantum Physics, Collaborative Innovation Center of Quantum Matter and Department of Physics, Tsinghua University, Beijing 100084, China; 5 Collaborative Innovation Center of Advanced Microstructures, Nanjing University, Nanjing 210093, China

## Abstract

Recently, the layered semimetal WTe_2_ has attracted renewed interest owing to the observation of a non-saturating and giant positive magnetoresistance (~10^5^%), which can be useful for magnetic memory and spintronic devices. However, the underlying mechanisms of the giant magnetoresistance are still under hot debate. Herein, we grew the stoichiometric and non-stoichiometric WTe_2_ crystals to test the robustness of giant magnetoresistance. The stoichiometric WTe_2_ crystals have magnetoresistance as large as 3100% at 2 K and 9-Tesla magnetic field. However, only 71% and 13% magnetoresistance in the most non-stoichiometry (WTe_1.80_) and the highest Mo isovalent substitution samples (W_0.7_Mo_0.3_Te_2_) are observed, respectively. Analysis of the magnetic-field dependent magnetoresistance of non-stoichiometric WTe_2_ crystals substantiates that both the large electron-hole concentration asymmetry and decreased carrier mobility, induced by non-stoichiometry, synergistically lead to the decreased magnetoresistance. This work sheds more light on the origin of giant magnetoresistance observed in WTe_2_.

Magnetoresistance (MR) is the change of electrical resistance under the application of a magnetic field. The materials with large MR can generate great potential applications in magnetic sensors^1^, magnetic information storage^2^, and so on. Recently, giant (~1.5 × 10^5^% at 2 K and 9 Tesla magnetic field) and non-saturated MR is observed in two-dimensional (2D) layered transition-metal dichalcogenides WTe_2_^3^. It leads to a series of works to study the novel physical properties of WTe_2_, such as superconductivity with *T*_*c*_ as high as 7 K under external mechanical pressure^4^, and possible quantum spin Hall effect in monolayer WTe_2_ with bulk electronic energy band-gap as large as 100.0 meV^5^. Certainly, the origin of extremely large MR is not only an important physical problem, but also valuable to device application of WTe_2_. As it was proposed, the main physical origin of giant MR is attributed to the nearly perfect compensation of electron and hole pockets^3^. This opinion is supported by subsequent Fermi surface determination by angle-resolved photoemission spectroscopy (ARPES)^6^, as well as suppressed MR under external mechanical pressure^7^.

However, recent work based on detailed ARPES claims that there are more subtle details in electronic band structure and abstract Fermi surface morphology in WTe_2_^8^. Quantum oscillation of MR (Shubnikov-de-Haas oscillation) substantiates that there are multiple fermion pockets^9^. Some studies find evidences for spin-orbit split bands in WTe_2_. Spin-orbit split bands suppress the inter- and intra-band backscattering^10^. In addition, a latest study implies that the large MR in WTe_2_ also may be related to the crystal quality or carrier mobility^11^, and the more apparent decreased effect of MR to aliovalent doping (Re and Ta) over simple isovalent substitution (Mo-doping) and the different growth method also support it^12^. The physical origin of extremely large MR observed in WTe_2_ therefore is still an open question.

In this work, considering the above-mentioned confusion, we intentionally introduce the non-stoichiometry and isovalent doping Mo in WTe_2_ to investigate the dependence among electron-hole asymmetry, carrier mobility and MR. Our systematic MR and Hall effect measurements substantiate the extremely large MR in stoichiometric WTe_2_. But large MR is disappeared in non-stoichiometric and isovalent Mo substitution WTe_2_ crystals. Based on analysis of magnetic-field dependent MR, both enlarged electron-hole concentration asymmetry and decreased mobility synergistically lead to the decreased MR in doped WTe_2_. From the viewpoint of real application, our result suggests that significant MR is strongly dependent on the stoichiometry of WTe_2_.

## Results

**Figure 1:**
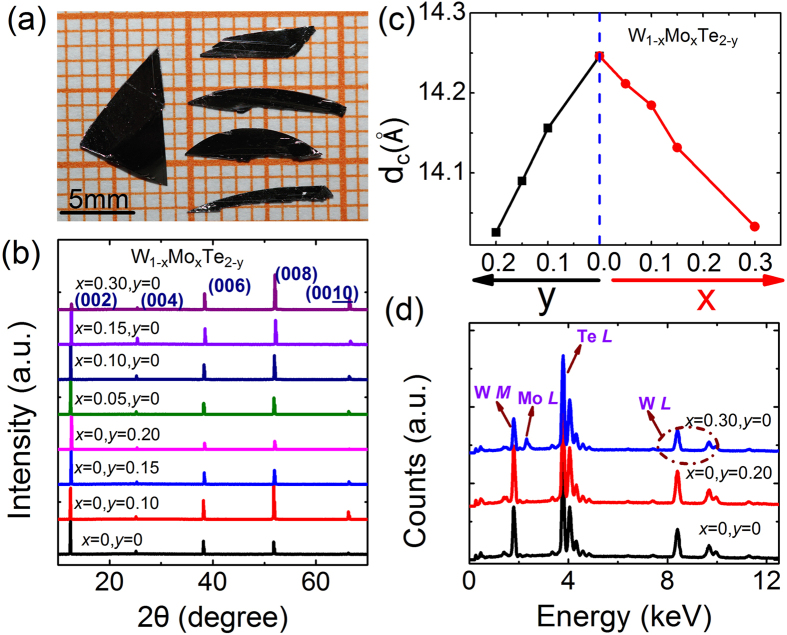
(**a**) The representative optical micrograph of the as-grown W_1−*x*_Mo_*x*_Te_2−*y*_ single crystals. (**b**) The XRD patterns of the single crystal W_1−*x*_Mo_*x*_Te_2−*y*_ samples. (**c**) The c-axis lattice parameter *d*_*c*_ as a function of Mo-substituting level *x* and non-stoichiometric level *y*, respectively. (**d**) The EDS spectra of three representative samples (WTe_2_, WTe_1.80_ and W_0.7_Mo_0.3_Te_2_).

The optical micrograph of the synthesized crystals is shown in Fig. 1(a). The crystals show metallic luster and sheet-shape morphology. And the largest sample can be reached to 9 × 5 × 0.5 mm^3^. The XRD patterns of single crystal W_1−*x*_Mo_*x*_Te_2−*y*_ samples are depicted in Fig. 1(b). Only the (00*l*) reflections are observed in these curves. It suggests that the exposed surface of crystals (see Fig. 1(a)) belongs to *ab*-plane and the thinnest dimension is along the *c*-axis. To show the effect of Mo substituted and non-stoichiometric on the lattice constant clearly, we plot the *c*-axis lattice parameter *d*_*c*_ for all W_1−*x*_Mo_*x*_Te_2−*y*_ samples in Fig. 1(c). It displays that the *c*-axis lattice parameter *d*_*c*_ decreases monotonically with increasing *y* or *x*. As a result, we suggest that the Mo^4+^ ions enter into W-sites, due to the ionic radius of Mo^4+^ ions (0.65 Å) being smaller than that of W^4+^ (0.66 Å), which gives rise to the decrease in the lattice parameter *d*_*c*_^13^. By the same mechanism, the lack of Te element also can lead to the decrease of the lattice parameters (see the black line of Fig. 1(c)). Figure 1(d) plots the EDS spectra of three representative samples of WTe_2_, WTe_1.80_ and W_0.7_Mo_0.3_Te_2_ crystals. These results prove that the samples with varied chemical composition are obtained.

**Figure 2:**
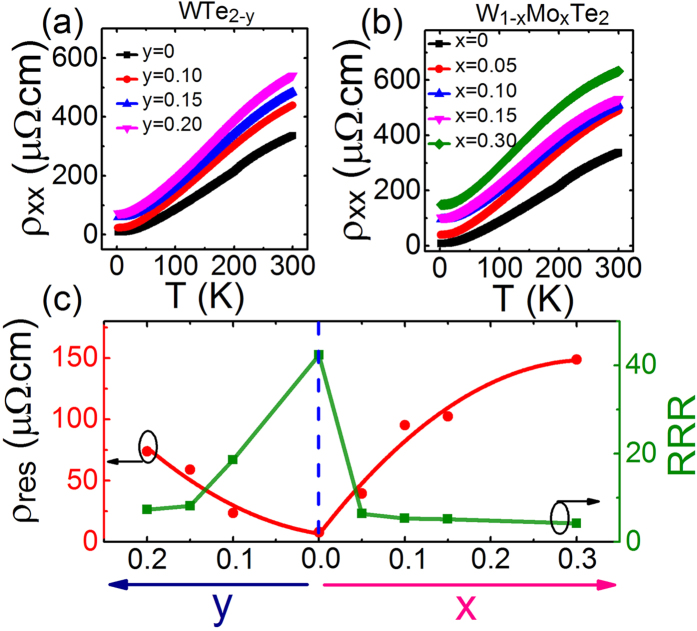
(**a**) The temperature-dependent *ab*-plane resistivity *ρ*_*xx*_ (from 2 K to 300 K) of the stoichiometric and non-stoichiometric WTe_2_ crystal samples. (**b**) The temperature-dependent resistivity *ab*-plane *ρ*_*xx*_ (from 2 K to 300 K) of the stoichiometric and Mo substituted WTe_2_ crystals. (**c**) The residual resistance *ρ*_*res*_ (red line) and the RRR value (green line) as a function of Mo-substituting level *x* and non-stoichiometric level *y*, respectively.

Figure 2(a) depicts the typical temperature-dependent *ab*-plane resistivity *ρ*_*xx*_, measured from 2 K to 300 K, for the stoichiometric and non-stoichiometric WTe_2_ crystals. They all show the metallic behavior but with different residual resistance. Quantitatively, the residual resistance of WTe_2_, WTe_1.90_, WTe_1.85_ and WTe_1.80_ are 8.0 × 10^−6^, 5.4 × 10^−5^, 5.9 × 10^−5^ and 7.4 × 10^−5^ Ω·cm, respectively. One can see that stoichiometric WTe_2_ sample has the minimum residual resistance. It infers that non-stoichiometry does induce the high density of defects/impurities, which in turn increases the residual resistance in non-stoichiometric WTe_2_ crystals^14^. The temperature-dependent resistances of stoichiometric and Mo substituted WTe_2_ crystals are presented in Fig. 2(b). As the same as the non-stoichiometric WTe_2−*y*_, they also demonstrate the metallic behavior and raising the Mo-substituting concentration increases the residual resistance of W_1−*x*_Mo_*x*_Te_2_ samples too. The relationship between the residual resistance *ρ*_*res*_, as well as the residual resistivity ratio (RRR) and Mo-substituting concentration *x* and non-stoichiometric concentration *y* are presented in Fig. 2(c). It substantiates that upon raising the Te deficiency and the Mo concentration, the density of defects/impurities in non-stoichiometric WTe_2_ samples gradually increases.

**Figure 3:**
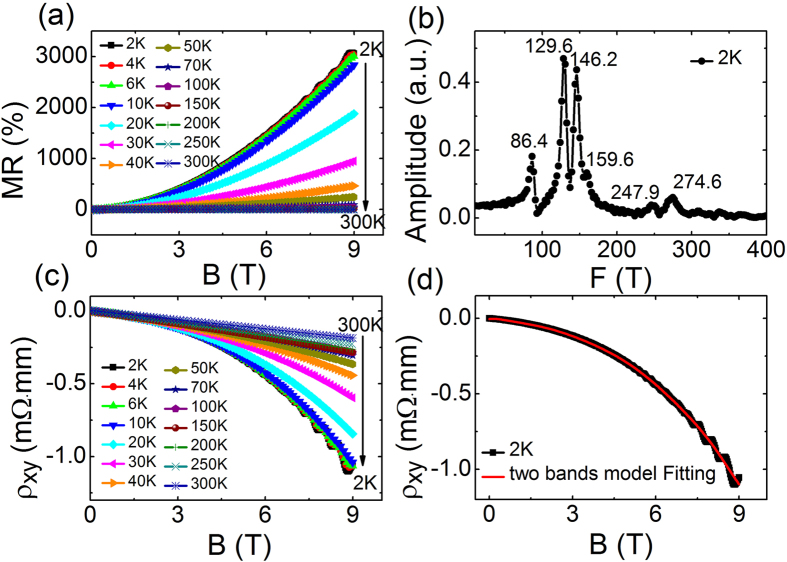
(**a**) The relationship between magnetoresistance (MR) and magnetic field of stoichiometric WTe_2_ single crystals under several temperatures, with magnetic field applied along the *c*-axis and scanned from 0 to 9 T. (**b**) Fast Fourier Transform (FFT) spectra at 2 K. The six major frequencies are observed. (**c**) The relationship between Hall resistivity (*ρ*_*xy*_) along *ab*-plane of stoichiometric WTe_2_ crystal and magnetic-field measured at variable temperatures. (**d**) *ρ*_*xy*_–B curve measured at 2 K and the corresponding fitting by two-band model.

In order to study the influence of the chemical composition changes on the MR property of WTe_2_, the transport properties of stoichiometric WTe_2_ crystals were investigated firstly. Figure 3(a) shows the curves of MR *vs* external magnetic field for WTe_2_ crystals under variable temperatures. The MR is defined as


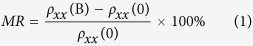


where *ρ*_*xx*_ and B are longitudinal resistance and magnetic field, respectively. One can see that curves of the MR are parabola-like up to 9 T. And the most impressive feature is that the MR of stoichiometric WTe_2_ reaches around 3100% at 2 K and 9 T. Though this value is smaller than that in the first paper reporting the giant MR (~10^5^%) in WTe_2_^3^, it is quite comparable to results in other reports^4^,^15^,^16^. As can be seen in Fig. 3(a), distinct Shubnikov-de-Haas oscillations are observed in MR-magnetic field curve measured at 2 K, which is related to the quantum behavior of the electrons or holes. And it is inferred that there is high enough carrier mobility in this crystal to observe the quantum oscillation^17^. After subtracting the smooth background of the MR measured at 2 K, the fast Fourier transform (FFT) analysis was carried out on the Shubnikov-de-Haas oscillations^18^. As shown in Fig. 3(b), we have identified four peaks: F_1_ ≈ 86.4 T, F_2_ ≈ 129.6 T, F_3_ ≈ 146.2 T, and F_4_ ≈ 159.6 T, which are consistent with the previous results^9^. In accordance with previous analysis, the oscillations of F_1_ and F_4_ come from hole, while the left two oscillations do electrons^9^. The observed peaks of 247.9 T and 274.6 T can be attributed to sum frequencies F_1_ + F_4_ and F_2_ + F_3_, respectively. Accordingly, by assuming isotropic parabolic dispersion, the electron and hole’s concentrations can be extracted as 3.2 × 10^19^ cm^−3^ and 3.7 × 10^19^ cm^−3^, respectively. To quantify the degree of the mismatch of electron and hole concentrations, we define the electron-hole concentration asymmetry factor *k* as


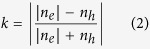


where *n*_*e*_ and *n*_*h*_ represent the electron and hole concentration, respectively. The small dimensionless value of asymmetry factor *k* represents the small mismatch between the electron and hole concentrations. The value of *k* in as-grown stoichiometric WTe_2_ can be calculated as 0.0725.

To further support above discussion, magnetic-field dependent Hall resistivity (*ρ*_*xy*_) is presented in Fig. 3(c) at variable temperatures. The negative and linear magnetic-field-dependent *ρ*_*xy*_ at high temperature (above 50 K) reveals that the dominant carrier is electron. According to the formula *R*_H_ = B/*ne* (where *R*_H_, *n*, and *e* represent the Hall resistivity^14^, carrier concentration and electron charge, respectively), the electron carrier concentration of WTe_2_ at 300 K is 3.0 × 10^20^ cm^−3^. But with the temperature decreasing, the dependence of *ρ*_*xy*_ on magnetic field obviously deviates from the linear relationship (below around 50 K). This suggests that hole and electron carriers contribute together for the electrical transport in WTe_2_ crystal. This is consistent to the result of the Shubnikov-de-Haas oscillation. So a classical two-band model was used to fit the non-linear relationship between Hall resistivity and magnetic field at 2 K (Fig. 3(d)). In two-band model, the Hall resistivity is described as^14^:


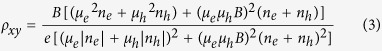


where *μ*_*e*_ and *μ*_*h*_ are the carrier mobility of electron and hole, respectively. Here, with the best fitting shown in Fig. 3(d), the values of electron and hole mobility are 4.0 × 10^3^ cm^2^V^−1^s^−1^ and 5.2 × 10^3^ cm^2^V^−1^s^−1^, respectively. In the fitting, the electron and hole concentrations extracted from Shubnikov-de-Haas oscillation are used. It should be noted that below around 50 K, the Hall curves deviate from the linear relationship gradually. Simultaneously, the MR is increased gradually up to 3100% at 2 K. It strongly suggests that extremely large MR is coincident to high carrier mobility and comparable concentration of electron and hole at low temperatures.

**Figure 4:**
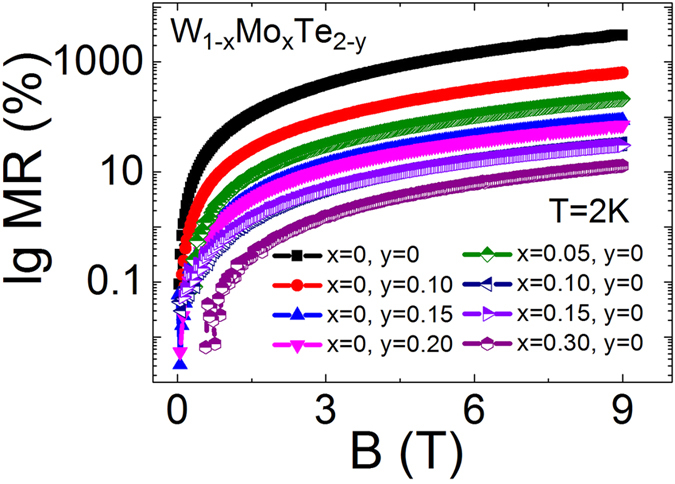
The relationship between MR and magnetic field in WTe_2−*y*_ (*y* = 0, 0.10, 0.15, 0.20) and W_1−*x*_Mo_*x*_Te_2_ (*x* = 0, 0.05, 0.10, 0.15, 0.30) crystals measured at 2 K.

The non-stoichiometric and Mo substituted WTe_2_ samples provide a platform to adjust the Fermi level and enhanced impurity scattering. It in turn affects the mismatch degrees of the electron and hole concentrations, as well as carrier mobility^14^. Compared with the stoichiometric WTe_2_ single crystals, the MR of non-stoichiometric WTe_2−*y*_ (*y* = 0.10, 0.15, 0.20) and Mo substituted WTe_2_ W_1−*x*_Mo_*x*_Te_2_ (*x* = 0.05, 0.10, 0.15, 0.30) single crystals is smaller (Fig. 4). Quantitatively, the MR of WTe_2−*y*_ (*y* = 0.10, 0.15, 0.20) and W_1−*x*_Mo_*x*_Te_2_ (*x* = 0.05, 0.10, 0.15, 0.30) measured at 2 K and 9 T magnetic field are 640%, 86%, 71%, 210%, 33%, 31% and 13%, respectively.

Here we analyze the physical origin of MR evolution in stoichiometric and non-stoichiometric WTe_2_ crystals. According to the two-band model with electrons and holes, the MR can be written as^14^:


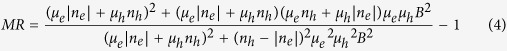


The meanings of symbols are the same as those described above. For simplifying this complex equation, we assume that electron and hole have same carrier mobility, so *μ*_*e*_ = *μ*_*h*_ = *μ* (*μ* is the mean mobility). This approximation is often used to describe the MR in the semimetals^19^. Thus, Eq. (4) can be simplified as


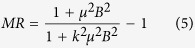


**Figure 5:**
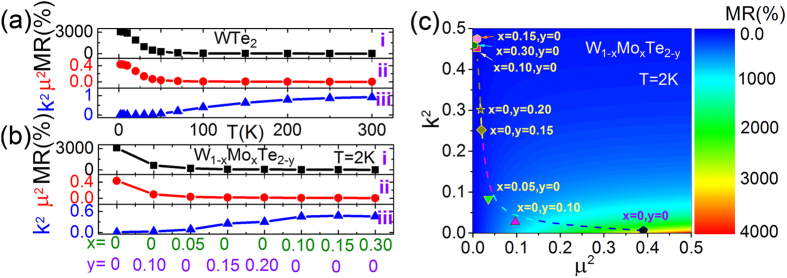
(**a**) Temperature dependence of MR (i), squared mobility *μ*^*2*^ (*μ* ≈ *μ*_*e*_ ≈ *μ*_*h*_, the unit of μ is m^2^V^−1^s^−1^) (ii), and squared electron-hole concentration asymmetry factor *k*^2^ 
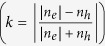
 (iii) for stoichiometric WTe_2_ crystal (shown in Fig. 4(a)). (**b**) The MR (i), *μ*^*2*^ (ii), and *k*^2^ (iii) for each composition W_1−*x*_Mo_*x*_Te_2−*y*_ (shown in Fig. 4). (**c**) The dependence of MR on *k*^2^ and *μ*^*2*^ at 2 K calculated by 
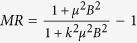
. Colored spots are experimental data extracted from Fig. 5(b).

So we firstly used this formula to fit all the curves at various temperatures and get the value of asymmetry factor *k* and the mean mobility *μ* for stoichiometric WTe_2_ (shown in Fig. 3(a)), summarized in Fig. 5(a). One can see that the mobility *μ* remains fairly steady below 10 K but gradually decreases with increasing the temperature above 20 K. On the contrary, the asymmetry factor *k* has a reverse trend. With the decrease of *μ* and the enlarging of *k*, the MR of stoichiometric WTe_2_ gradually decreases. Thus we speculate the large MR effect in WTe_2_ material may be related to both electron-hole concentration asymmetry and the carrier mobility. In order to verify the assumption, we further used the equation (5) to fit all the curves at 2 K and get the value of asymmetry factor *k* and the mean mobility *μ* for each composition W_1−*x*_Mo_*x*_Te_2−*y*_ (shown in Fig. 4). As depicted in Fig. 5(b), upon raising the lack of Te element or increasing Mo-substituting concentration, the electron-hole concentration asymmetry enlarges and carrier mobility decreases. Both these two factors lead to the decreased MR in non-stoichiometric and Mo substituted WTe_2_. Figure 5(c) shows the asymmetry factor *k* and the mean mobility *μ* dependent MR colored picture, which is calculated from the equation (5). As shown in Fig. 5(c), one can find that the highest MR is observed at the bottom-right region with the equal electron-hole concentration and the largest mobility. In the real material systems, the colored discrete points of each composition distributes along the dash line in Fig. 5(c). Thus, one can conclude that electron-hole concentration asymmetry, induced by both non-stoichiometric and Mo-substituting, leads to the dramatically depressed MR in WTe_2_ system. Furthermore, the new lesson we learned here is that decreased carrier mobility also can lead to the depressed MR. Quantitatively, there is only 13% MR in W_0.7_Mo_0.3_Te_2_. In this case, the corresponding electron-hole concentration asymmetry (*k*) and mobility (*μ*) are 0.68, 5.7 × 10^2^ cm^2^V^−1^s^−1^, respectively. Compared with stoichiometric WTe_2_ crystal, *μ* of W_0.7_Mo_0.3_Te_2_ is much smaller than stoichiometric one. Thus it suggests that both electron-hole concentration and carrier mobility play the crucial role on the MR in WTe_2_ material.

Here we’d like to compare aforementioned work to current available similar works. Flynn, *et al*. also found that the significant MR in 1% Mo, Re or Ta doped WTe_2_ is nearly lost, which is attributed to the large electron-hole asymmetry induced by Re or Ta-doping^12^. But it can be seen that the specimens in this work are ceramic ones. We suspect that the low carrier mobility in ceramic samples also contributes the decreased MR therein. Ali, *et al*. compared the MR of WTe_2_ synthesized by both chemical vapor transport and self-flux methods^11^. It claimed that WTe_2_ synthesized by self-flux method has larger mobility than one by chemical vapor transport, which leads to larger MR in WTe_2_ synthesized by flux method. Compared these works to current one, it is obvious that based on our systematic single crystals W_1−*x*_Mo_*x*_Te_2−*y*_ samples, the relationship among MR, electron-hole asymmetry and carrier mobility is clearly revealed.

## Conclusions

In summary, we intentionally synthesized the non-stoichiometric and Mo substituted WTe_2_ single crystals to study the effect of electron-hole concentration asymmetry on the MR of WTe_2_. It is substantiated that no matter in non-stoichiometric or in Mo substituted WTe_2_ single crystals, there is dramatically decreased magnetoresistance. The quantitative magnetoresistance fitting substantiates that non-stoichiometric and Mo-substituting not only induces the electron-hole concentration asymmetry, but also generates the decreased mobility. These two factors *synergistically* lead to the dramatically decreased magnetoresistance in non-stoichiometric WTe_2_ crystals. Thus, it is crucial to obtain high purity single samples to realize the equal amount of electrons and holes, as well as high carrier mobility. Our work will provide an important clue for the exploring of such large MR materials.

## Methods

Single crystals of W_1−*x*_Mo_*x*_Te_2−*y*_ were grown by a chemical vapor transport method using Br_2_ as the transport agent. All polycrystalline samples of W_1−*x*_Mo_*x*_Te_2−*y*_ were synthesized from high purity elemental powders W (99.99%), Mo (99.99%) and Te (99.999%) by solid state reaction in evacuated quartz tubes. It is worthwhile to mention that *in nominal stoichiometric WTe*_*2*_ *sample described below, the mole ratio of W and Te in raw materials is set as 1:2*. Afterwards, mixtures of as-prepared polycrystalline W_1−*x*_Mo_*x*_Te_2−*y*_ and Br_2_ (about 5 mg/L) were loaded into the sealed evacuated quartz tube, and then placed at a double zone furnace with a temperature gradient between hot end 850 °C and cold end 750 °C to grow crystals for 10 days. Then all the crystals samples were characterized by X-ray diffraction (XRD) measurement in an X-ray diffractometer (Ultima III Rigaku) using Cu-*K*α radiation with 2θ scanned from 10° to 70°. The detailed elemental compositions of the as-grown crystals were determined by a scanning electron microscope (SEM, FEI-Quanta) equipped with an energy-dispersive spectroscopy (EDS) spectrometer. Standard six-probe method was used for the electrical resistivity, MR, and Hall resistance measurements. These characterizations were performed in a 9 T physical properties measurement system (PPMS, Quantum Design). The magnetic field was perpendicular to the *ab*-plane in our magneto-transport measurements.

## Additional Information

**How to cite this article**: Lv, Y.-Y. *et al*. Dramatically decreased magnetoresistance in non-stoichiometric WTe_2_ crystals. *Sci. Rep*. **6**, 26903; doi: 10.1038/srep26903 (2016).
